# Consumer Intention towards Buying Edible Beef Offal and the Relevance of Food Neophobia

**DOI:** 10.3390/foods12122340

**Published:** 2023-06-11

**Authors:** Maria Sabbagh, Luciano Gutierrez, Roberto Lai, Giuseppe Nocella

**Affiliations:** 1 Department of Agricultural Sciences, University of Sassari, 07100 Sassari, Italy; lgutierr@uniss.it; 2 Cooperativa Produttori Arborea—Società Agricola, 09092 Arborea, Italy; rlai@uniss.it; 3 Department of Applied Economics and Marketing, School of Agriculture, Policy and Development, University of Reading, Reading RG6 6UR, UK; g.nocella@reading.ac.uk

**Keywords:** edible offal, food neophobia, food disgust sensitivity, theory of planned behaviour, consumption

## Abstract

Enhancing the willingness to eat edible offal can be a valuable strategy to mitigate the greenhouse gas (GHG) emissions related to growing meat production and to provide food with high protein content to a growing global population. Although some edible offal is considered delicacies, we hardly find such foods in Western countries’ everyday diet, and their human consumption has decreased during the last decades. This study analyses the consumer purchase intention of BEEF edible offal using an extended version of the Theory of Planned Behaviour (TPB), where food neophobia and food disgust sensitivity play an essential role in determining consumers’ willingness to eat beef edible offal. An online survey was conducted among a sample of Italian adult regular meat eaters (*n* = 720), stratified by age, gender, education and residence. The results showed a direct negative impact of food neophobia on the intention to consume offal. Further, we were able to quantify a negative indirect impact of food neophobia on intention through the mediation of food disgust sensitivity and attitudes, subjective norms and perceived behavioural control, which all exert an essential role in determining the willingness to consume beef edible offal. We found that the mediated impact of food neophobia on the intention to consume beef offal is much higher than the direct impact. In conclusion, recommendations and implications, such as promoting cooking shows with celebrity chefs, new products or new packaging of edible offal, were developed based on the results to increase edible beef consumption.

## 1. Introduction

In the next decade, the global meat supply is expected to increase. One of the primary sources of greenhouse gas (GHG) is related to the cattle production system, with 64–78% of all global anthropogenic emissions [1,2]. Additionally, the GHG impact on the environment of the meat sector is projected to rise by 9% by 2031 [3]. In this framework, increasing the consumption of animal by-products could be a win-win mitigation strategy because it will imply the reduction of GHG emissions through minor needs of livestock production and will consent to feed a growing global population by satisfying the demand for food with high protein content, e.g., [2,4,5,6]. In other words, this strategy will make be possible to obtain the most significant value from low-used resources and to reduce livestock’s negative impacts on the environment [7].

Animal by-products, also defined as the fifth quarter or offal, are generally defined as the non-meat parts of a carcass [7]. They can account for more than 44% of beef animal live weight, depending on the gender, species, and age of the animal [8]. Different terms and classifications are associated with animal by-products depending on countries’ regulations. In the United States, animal by-products are categorized as edible offal, inedible offal, and hides. Edible offal, also known as red offal, is mainly constituted of the kidney, heart, tongue, lung, stomach, spleen, liver, and tail. In this country, inedible offal, or white offal, is mainly represented by the lung, pancreas, and oesophagus [2]. In Europe, under Regulation (EC) 1969/2009, animal by-products are classified into three categories according to the risk they pose to the public. Categories 1 and 2 comprise all body parts of animals that are not edible due to the high or medium risk associated with pathologies and diseases. Category 3 animal by-products are usually considered at low risk and include carcasses and parts of a slaughtered animal that, although suitable for human consumption, are not channelled into the food chain for commercial reasons. Thus the current European categorization helps to define animal by-products more from a waste management point of view than from a food chain perspective [2].

The scope of the paper is to investigate the psychological and behavioural factors behind the intention to consume beef edible offal. As is well known, offal contains relevant amounts of nutrients such as vitamins, minerals, low-fat levels, and essential amino acids that make them nutritious food [9,10]. However, although certain offal is considered delicacies, edible offal is a relatively rare food in the everyday diet in many parts of the world. In the last decades, the expenditures for human consumption have decreased in many countries [11]. Thus, scarce consumers’ demand for edible offal may need to be revised to ensure what may seem like a practical policy strategy, i.e., increasing their consumption and avoiding treating offal mainly as a waste with negative environmental and cost repercussions [6,12].

Only a few studies have analysed consumer perceptions and willingness to eat edible offal [4,13,14]. Ref. [13] found that the perception of consumers on edible offal is analogous to the following factors that are usually considered important in purchasing red meat: price, freshness, visual display and packaging. Ref. [14]’s study suggested that sensory, safety and healthiness perceptions of offal were behind the negative attitude toward consumers’ offal choice. Ref. [4], using hierarchical linear regression, investigated the importance of food neophobia, food disgust and sociodemographic variables on the willingness to engage with animal by-products. They found that food neophobia and food disgust are important factors conditioning the consumer acceptance of animal by-products.

To analyse the consumer purchase intention of edible offal, we used the well-established Theory of Planned Behaviour (TPB), which identifies human action as a combination of the following three constructs: attitudes, subjective norms and perceived behavioural controls [15]. In recent years, there has been an increased application of the TPB to understand meat-eating behaviour [16,17,18]

In this study, we analysed the extent to which the TPB constructs (attitudes, subjective norms and perceived behavioural control) influence the willingness to consume beef edible offal. We extended the TPB model to include nutritional-psychological variables such as food neophobia and personal food disgust sensitivity, which were both reported to be relevant to people’s willingness to consume offal [2,19,20].

## 2. Background

To develop the conceptual framework, we adopted one of the most widely used methodologies for analysing human behaviour, i.e., the Theory of Planned Behaviour (TPB) by [15]. The predictive power of the TPB was validated by meta-analysis research, e.g., [21,22,23,24] and has been extensively used to analyse meat-eating behaviour in recent years [16,17,18]. Attitudes embody the degree to which a behaviour is considered positive or negative; subjective norms refer to the perceived social pressure from other individuals as colleagues and friends or relatives, to perform the behaviour, while perceived behavioural control collects the easiness or difficulty in performing the specific behaviour.

The literature suggests that other factors can influence behavioural intentions. In our work, mediating factors could be related to nutritional-psychological variables such as food neophobia and food disgust sensitivity [2,4].

### Food Neophobia and Food Disgust Sensitivity

Individuals’ sociodemographic attributes and product characteristics have often been identified as the main determinants of consumers’ choices of novel food, e.g., [2,19]. Humans’ choice of novel foods has been frequently related to a process labelled “omnivore’s paradox”, which describes the choice for these foods as a relentless tension between seeking novelty and variety (neophilia) and the aversion toward novel or unfamiliar foods (neophobia) [19,25,26]. This tension was found especially relevant among humans in response to eating animal products compared to non-animal products [26,27,28]. Studies have usually addressed the importance of food neophobia more than neophilia, suggesting a negative relationship between food neophobia and consumption of unfamiliar food, e.g., [20,29,30].

Consumption of edible offal is now less common in the Western diet, and people with a high score on food neophobia were less inclined to eat these products [2,4,6]. Sociodemographic variables seem to affect food neophobia [20,26,31,32,33]. According to these studies, younger people may be less affected by food neophobia because they have a higher exposure to novel food and/or a much broader food selection than older people determining a positive relationship between age and food neophobia. Further, neophobia seems to decrease as education increases [33,34,35] and as income increases [33]. While the effect of gender on food neophobia, some studies found significant gender differences, with more neophobic men than women [34,35]. However, other studies report no significant gender differences [26,32].

Food disgust sensitivity, also labelled as *the behavioural immune system* [36], refers to the refusal of foods considered potentially harmful to health [37,38]. Ref. [4] found in their study that for some consumers, animal offal has a negative association mainly related to disgust, confirming the expectations of [2] regarding the importance of sensory qualities for the individuals’ choice to consume these products.

Food disgust sensitivity has been reported to be influenced by the individual food neophobia level. For instance, [39] found a positive and significant correlation between food neophobia and disgust; [40] showed that food neophobia directly affects food disgust sensitivity.

## 3. The Present Study

In light of the literature reviewed above, the present study investigated the impact of TPB constructs (attitudes, subjective norms, perceived behavioural controls), food neophobia and food disgust on the intentions to consume beef edible offal considering the influence of sociodemographic variables. By the TPB model and previous studies [14], we hypothesised that attitudes, subjective norms and perceived behavioural control significantly and positively influence behavioural intention to consume beef edible offal. Specifically, we made the following hypotheses:

**Hypothesis** **1 (H1).** *The attitude would significantly predict a positive intention to consume edible beef offal.*

**Hypothesis** **2 (H2).** *Subjective norms would significantly predict a positive intention to consume edible beef offal.*

**Hypothesis** **3 (H3).** *Perceived behavioural control would significantly predict a positive intention to consume edible beef offal.*

We are not aware of other studies which analysed the influence of food neophobia and food disgust on attitudes, perceived behavioural control, subjective norms and willingness to consume beef edible offal. Some studies have reported that individuals with higher food neophobia have more negative attitudes and a lower willingness to eat cultured meat, insects and ethnic food, e.g., [29,30,41,42,43,44].

We test the following hypotheses that food disgust sensitivity mediates the influence of food neophobia on attitudes, social norms and perceived behavioural control:

**Hypothesis** **4 (H4).** *Perceived food disgust would significantly predict negative attitudes.*

**Hypothesis** **5 (H5).** *Perceived food disgust would significantly predict negative subjective norms.*

**Hypothesis** **6 (H6).** *Perceived food disgust would significantly predict a negative perceived behavioural control.*

We test the hypothesis that food neophobia directly and negatively influences the behavioural intention to consume beef edible offal:

**Hypothesis** **7 (H7).** *Perceived food neophobia would significantly predict a negative intention to consume edible beef offal.*

Further, following [45], we tested the hypothesis that food neophobia positively influences food disgust sensitivity.

**Hypothesis** **8 (H8).** *Food neophobia would significantly predict positive perceived food disgust sensitivity.*

**Figure 1:**
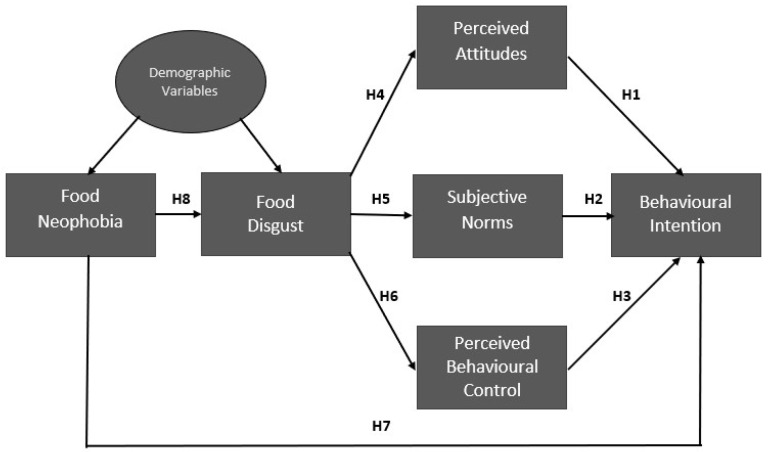
The hypothesised model.

Finally, we allow demographic variables to influence food neophobia and food disgust sensitivity. The hypothesized model is presented in Figure 1.

## 4. Methods

### 4.1. Sample and Procedure

The data were collected through an online survey using Qualtrics Platform conducted among Italian consumers with the help of an external data collection agency. Informed consent at the beginning of the questionnaire indicated the objective of the research and that participants could enter it voluntarily and anonymously. The questionnaire, previously tested among a sample of 50 participants that gave rise to minor changes, was carried out during the week of 16 November 2022–25 November 2022. Participants were randomly stratified by age, education, gender and residence area to reflect the Italian population. A total of 1058 questionnaires were collected, and 135 were incomplete. The Inclusion criteria were implemented so that individuals that are not regular meat eaters (203 participants, 22%) were excluded from the analysis. A total of 720 completed questionnaires were available for the analysis for adults (≥18 years of age). Thus, the sample size is larger than the minimum recommended sample size of 444 for an expected size effect of 0.3, 6 latent variables, 43 observed variables, *p*-value equal to 0.05 and statistical power of 0.9, as suggested by [46] for SEM models.

### 4.2. Measures

Below we present the measures utilized in the study shown in Figure 1.

#### 4.2.1. Intention

Intention to consume edible beef offal was measured with two items (IN1 “I intend to consume beef edible offal in the next future”; IN2 “I will recommend other people to consume edible beef offal in the next future”) using a 5-point Likert scale from (1) strongly disagree to (5) strongly agree.

#### 4.2.2. Attitude

Four items assessed the attitude towards consuming edible beef offal (I would eat beef edible offal because…AT1: “I like to eat them”; AT2: “they are a cheap source of protein”; AT3: “food waste can be reduced by this”; AT4: “they are healthier than other types of meat”). Each item was presented in the form of a 5-point Likert scale (e.g., 1 = strongly disagree, 5 = strongly agree). These items were adapted from the previous literature, see [4,14].

#### 4.2.3. Subjective Norms

Subjective norm was measured through two items (SN1: “Among the people close to me, I notice an interest in the consumption of edible beef offal”, SN2: “My family would be happy if I bought edible beef offal”) on a 5-point Likert scale from 1 strongly disagree to 5 strongly agree.

#### 4.2.4. Perceived Behavioural Control

Perceived behavioural control was measured through three items (PBC1: “I know butchers and/or supermarkets where I can buy edible beef offal”, PBC2: “I know restaurants where I can eat dishes based on offal”, PCB3: “I know how to cook beef edible offal-based foods”). They were measured using a 5-point Likert scale (e.g., 1 = strongly disagree to 5 = strongly agree).

#### 4.2.5. Food Neophobia

To measure the food neophobia scale (FNS), we use 8 of 10 items indicated in Pliner, P. and K. Hobden [26] (FN1: “I am constantly sampling new and different food” (R), FN2: “I don’t trust new foods”, FN3: “If I don’t know what is in a food, I won’t try it”, FN4: “Ethnic food looks too weird to eat”, FN5: “At dinner parties, I will try a new food” (R), FN6: “I am very particular about the foods I will eat”, FN7: “I am afraid to eat things I have never had before”, FN8: “I will eat almost anything” (R)) were translated in Italian. A 5-point Likert scale was also used, in this case, ranging from (1) “do not agree at all” to (5) “fully agree”. The items indicated with (R) were reversed. Concerning Pliner, P. and K. Hobden [26]‘s scale, two items were excluded (”I like food from different countries” and ”I like to try new ethnic restaurants”) to limit the maximum time for completing the questionnaire to 8 min.

#### 4.2.6. Food Disgust Sensitivity

Disgust was measured through a Food Disgust Scale [47], providing participants with five pictures and asking, “Closely look at this picture. Imagine you were asked to consume this food. Please indicate how disgusting you perceive this item to be”. The five pictures can be recovered from the Appendix A to this paper. The five items include pictures of (FD1: wrinkled tomatoes; FD2: beef tongue; FD3: marmalade with mould; F4: decaying banana; F5: hands handling dough, rings and painted nails). The pictures (presented in the Appendix A) consider different psychological effects related to possible human contamination, mould, decaying fruit and vegetables and animal flesh. Participants gave their answers on an interactive slider, which ranged from 0 to 100. As before, the number of pictures was restricted to five instead the 8 proposed by [47] to limit the maximum time for completing the questionnaire to 8 min.

### 4.3. Data Analysis

Partial least square-structural equation modelling (PLS-SEM) analysis was carried out to test the hypothesised model using Smart-PLS4 software [48,49]. This approach allows for a “causal-predictive” approach to structural equation modelling (SEM) [49]. We used to PLS-SEM instead of covariance-based SEM because we think our study is more on the explanative capacity of the components, such as food neophobia and food disgust, rather than a reproducing of the covariance matrix to achieve model fit. It seems that in this context, PLS-SEM is deemed more suited than covariance-based SEM [49,50].

In the data analysis, the indicator loading values, the internal consistence reliability (evaluated using the composite reliability indicator [51]), the convergent validity (Average Variance Extracted, AVE) and the discriminant validity (heterotrait-monotrait, HTMT, ratio of correlations [52,53]) of the measurement model were first examined. Before assessing the structural model validity, collinearity was analysed to make sure it did not bias the regression results. This task was completed by computing the Variance Inflation Factor (VIF). Ref. [54] suggests a VIF value lower than 3.3 exclude potential issues with collinearity problems. Structural model validity was evaluated by the Standardized Root Mean Square Residual (SRMR). We do not report other usually cited fit indicators such as RMSEA and χ(2) because of the scarce meaning in using them in the context of the PLS-SEM estimation method [49,55]. We also present the adjusted-R^2^ of the endogenous constructs and the f^2^ results of the measures [46]. Following [49,55], the PLS path model’s predictive accuracy was assessed by computing the Q^2^ value [56]. The Q^2^ is calculated by removing single points in the data matrix and substituting them with the means. The model is then re-estimated to assess the predictive accuracy of the data points that were removed. If the differences between the predicted and the original values are small, the Q^2^ will be higher, thereby indicating a higher predictive accuracy. Finally, we also analyse the predictive power of the structural model using Ref. [57]‘s prediction procedure provided by the Smart-PLS4 package. Because PLS-SEM is a distribution-free method, the statistical inference was made by using a bootstrapping procedure with 10.000 subsamples as suggested by [58], bias-corrected confidence interval (see Ref. [59]), two-tailed test type at the 5% level of significance.

## 5. Results

### 5.1. Sociodemographic Characteristics

The results of the sociodemographic information characteristics of the survey participants are presented in Table 1. The sample (N = 720) broadly reflects the adult populations in Italy according to the national Census (https://www.istat.it/en/population-and-households (accessed on 3 October 2022)). Females and males represented respectively 51% and 49% of the sample population distribution. Most participants had aged between (50 and 64) years (41.4%), had an income in the range of 20,000–30,000 euros, and 74.1% of the sample had a household size between 1 and 3 persons. On average, participants consumed meat during 2–4 days per week. Most participants stated to have a diploma in secondary education (45.8%), and post-secondary education (29.6%) is the second highest. The geographic distributions of the survey respondents see 45.7% of them in the North, 22.6% living in the Centre and 31.7% in the South and Islands.

### 5.2. Measurement Model

Table 2 show means and standard deviations for the single items (not presented for the constructs because PLS-SEM scales the scores to ensure that constructs have a mean of zero and a unit variance), the loadings and their confidence intervals, the Cronbach’s alpha, the composite reliability (CR) and average variance extracted indicators (AVE) of the constructs. As concerns the mean scores, participants showed a strong positive attitude towards the consumption of offal as a method to reduce food waste (M = 3.39, SD = 1.27). The respondents declared only a moderate level of social pressure to execute the buying behaviours, and they perceived offal as easy to buy because they know butchers and supermarkets where they are sold (M = 3.42, SD = 1.36). Cronbach’s and composite reliabilities coefficients showed that, in general, the constructs had high levels of internal consistency with acceptable values of Cα for the perceived behavioural control and food disgust sensitivity constructs. Similarly, the variables showed good convergent validity because the constructs present values of the AVE higher than 0.50 [49]. Further, the construct reliability coefficients ρ_A_ and ρ_C_ [60], not included for space, have values above 0.7.

In Table 3, we show that the discriminant validity assessment, based on Ref. [52]’s heterotrait-monotrait ratio of correlations measure, has values less than 0.85 supporting the measures’ discriminant validity.

### 5.3. Structural Model

Before assessing the structural relationships, the collinearity was examined to exclude the possible presence of bias in the regression results. We computed the VIF [54] values for the constructs (not reported for brevity). The indicator’s values were in the range [1.07,1.89], thus well below the threshold value of 3 [55].

Table 4 illustrates the outcomes of the path coefficient analysis and the adjusted R^2^. The Q^2^ indicator was above 0 for each endogenous construct suggesting that the model has predictive relevance and the SRMR well below the value of 0.08 suggested by [61]. Further, we also analyse the predictive power of the structural model using Ref. [57]’s prediction procedure provided by the Smart-PLS4 PLSpredict package. The value of Q^2^ indicators (not presented but available upon request) for the constructs are positive (with the exception of the subjective norms construct, which showed a value of Q^2^ = −0.003), meaning that the prediction error of the PLS-SEM model is smaller than the prediction error of simply using the mean values, i.e., the PLS-SEM model offers better predictive performance.

The results in Table 4 show that the elements of the TPB model, attitudes (β = 0.34, *p* < 0.01), subjective norms (β = 0.24, *p* < 0.01) and perceived behavioural control (β = 0.34, *p* < 0.01) have a positive and significant effect on the behavioural intention to consume beef edible offal. Differently, food neophobia (β = −0.08 *p* < 0.05) negatively impacts the intention to consume beef edible offal. The frequency of meat consumption during the week was not found significant in influencing the intention to consume offal, and this variable was not included in the path analysis.

Food disgust sensitivity negatively and significantly influenced attitudes (β = −0.47, *p* < 0.01), subject norms (β = −0.48, *p* < 0.05) and perceived behavioural control (β = −0.39, *p* < 0.01). Finally, food neophobia positively influences food disgust (β = 0.40 *p* < 0.01). Thus, people with higher food neophobia scores also have a higher food disgust sensitivity score.

This last result and those related to the impact of food disgust on attitude, subjective norm and perceived control imply that food neophobia influences the willingness to consume edible offal intention to consume offal mainly indirectly. The indirect effect (β = −0.24, *p* < 0.01) is negative and significant and much higher than the direct effect, which is equal to (β = −0.08, *p* < 0.05).

Age impacted significantly and negatively on food disgust sensitivity (β = −0.11, *p* < 0.01). Thus, older people seem to have a lower level of food disgust than younger people. Gender was also significant with a negative sign, which implies that women have a higher food disgust sensitivity than men (β = −0.09, *p* < 0.05). Similar results for age and gender were also found by [6]. People living in the South of Italy have a higher level of food disgust (β = −0.09, *p* < 0.05). Finally, income negatively influences food neophobia, indicating that participants who have lower neophobia scores are people with a higher level of income. The income variable influenced significantly and negatively food neophobia (β = −0.17, *p* < 0.01).

We assess the in-sample model fit considering the adjusted R^2^. We found the intention to consume offal has a value of R^2^ = 0.65, while the other endogenous constructs have a value around 0.20, which could be rather small, but given the complexity of the model, we consider it acceptable for the study.

As well, Ref. [46] indicated that f² has a small effect size if (≥0.02), medium if (≥0.15) and large if (≥0.35). We found that f² has a medium effect size regarding attitudes on behavioural intention (f^2^ = 0.19), disgust on attitudes (f^2^ = 0.28), perceived behavioural control (f^2^ = 0.18) and subjective norms (f^2^ = 0.30). Furthermore, the f² has as well a medium effect size with regards to neophobia on disgust (f^2^ = 0.20) and perceived behavioural control on behavioural intention (f^2^ = 0.18). Small effect sizes of f² are related to the neophobia on the behavioural intention (f^2^ = 0.02) and to the subjective norms on the behavioural intention (f^2^ = 0.10).

## 6. Discussion

Satisfying the growing demand for food with high protein content is today a global challenge, and making better use of offal could be a strategy that would be an effective policy for supplying food resources otherwise largely wasted. Moreover, this strategy would help reduce the environmental impact of growing meat production. Thus, policies that could enhance the willingness to consume low exploited offal can have beneficial effects on the environment and for the feeding of a growing global population.

This study used the TPB to understand adults’ psychosocial factors motivating the consumption of edible beef offal for a stratified sample of Italian adults. The TPB was extended to consider the effects of personal characteristic as food neophobia and food disgust. The results confirmed the importance of enlarging the model, including these variables, which allowed us to consider important aspects of the consumption of beef edible offal. Food neophobia and food disgust sensitivity have been found important in conditioning the consumption of novel foods by many previous works, e.g., [2,19,20]. The TPB accounted for 64.5% of the variance in willingness to consume edible offal, while the extended model explained 66.0% of the variance.

The results supported our H1, H2 and H3 hypotheses, showing that attitudes, subjective norms and perceived behavioural control explain a large share of the variance of the intention to consume edible offal. A study by [14] presented similar findings. In our model, perceived behavioural control exerted the most important impact, followed by attitudes and subjective norms, which exerted the weakest influence. The results support previous research that found subjective norms as a weaker predictor of the intention to consume cultured meat [62] or insects [63]. Ref. [64] showed that the effects of attitude and subjective norms on intention are moderated by perceived behavioural control, and a greater perceived behavioural control weakens the relative importance of subjective norms while strengthening the relative importance of attitude in the prediction of intention. Since the higher importance of perceived behavioural control found in our study, Ref. [64]’s results can explain the weaker effect of subjective norms found in our study.

Food disgust sensitivity was detected to negatively and significantly impact attitudes, perceived behavioural control and subjective norms. The strongest impact was found on attitudes.

Previous studies analysed the relationship between food neophobia and food disgust, showing that the two behaviours are positively linked, i.e., the higher the tendency to reject new foods (neophobia), the greater the food disgust in eating them will be [40]. Our results support this hypothesis. Thus, food neophobia exerted a significant indirect effect on the intention to consume beef edible offal through the mediation role of food disgust sensitivity. We were also able to find a direct effect of food neophobia on acceptance of consuming edible offal, but the impact is lower than the previous indirect effect through the mediation role of food disgust sensitivity.

Sociodemographic variables influence food neophobia and food disgust sensitivity. Contrary to Ref. [65]’s results and in line with Ref. [38]’s findings, our results support the idea that there is a constant decline in food disgust sensitivity over the life course. As a consequence, older people seem to have a lower level of food disgust sensitivity than younger people, and this result could be related to older people’s early life experiences with the consumption of offal. Females reported higher disgust sensitivity than males.

The level of income positively impacted food neophobia, indicating that participants who have lower neophobia scores are people who stated a higher level of income. This result has also been reported by other authors as Ref. [33].

## 7. Conclusions

Increasing and valorising the consumption of animal by-products may enhance the viability of the meat sector, which is under the competitive pressures of vegetal protein sources [2]. Heavy constraints in developing a strategy may derive from consumers’ attitudes and personal traits linked to food disgust sensitivity and food neophobia. Endorsing the approach developed by Ref. [66] and recommended by Ref. [2] for meat coproducts, interventions and policies devoted to changing ingrained behaviour patterns have to be linked to enhancing capabilities, opportunities and motivations of behaviour (COM-B model) related to a specific behaviour. Increasing capabilities will require allowing individuals to know more about the importance of eating offal. Tasting foods was indicated as one of the most efficient means to ensure neophobia reduction in novel foods [67]. Thus, in light of overcoming negative attitudes towards the consumption of edible beef offal, especially driven by neophobia and/or disgust reactions, it could be important to promote, for instance, tasting sessions, cooking shows involving experts and celebrity chefs [6]. These actions could convince non-offal eaters to accept them as food and feel less unconvinced and disgusted about consuming offal, given the important role exerted by friends in consumer concerns. Furthermore, to enhance motivations, a wider media coverage devoted to stressing the similarity to consuming beef edible offal or beef meat and that helps to emphasize the environmental and sustainability aspects of the consumption of offal may improve consumers’ motivations and intentions to eat edible offal. According to Ref. [2], enhancing opportunities that is all factors that lie outside the sphere of an individual’s decisions, will require the science and technologies interface to create opportunities for new products and/or new packaging and presentations of edible offal to consumers. Last but not least, letting consumers be aware of the nutritional value of offal should be one of the interventions, which will help to reduce consumers’ barriers towards wider eating of edible offal.

## Figures and Tables

**Table 1:** Demographic characteristics of individuals interviewed.

	Variable	Percentage	Frequency
Gender			
	Female	51.1	368
	Male	48.8	351
	Prefer not to say	0.1	1
Household size			
	1–3	74.1	535
	4–6	25.3	182
	>7	0.6	3
Household weekly meat consumption (days)			
	1–2	35.1	253
	3–4	47.9	345
	5–6	13.2	95
	7	3.8	27
Age			
	18–49	31.7	228
	50–64	41.4	298
	>65	26.9	194
Annual Income			
	<10.000 euro	12.1	87
	10.000–20.000 euro	22.9	165
	20.000–30.000 euro	25.0	180
	30.000–40.000 euro	17.1	123
	40.000–50.000 euro	11.9	86
	>50.000 euro	11	79
Educational Status			
	Less than Secondary	24.6	177
	Secondary	45.8	330
	Higher than Secondary	29.6	213
Regional Area			
	North	45.7	329
	Centre	22.6	163
	South and Islands	31.7	228

**Table 2:** Item mean and standard deviation, item loading and their confidence intervals, constructs Cronbach’s alpha(Cα), composite reliability (CR) and average variance extracted (AVE).

Constructs and Items	M	SD	Loading Values	Confidence Interval		Cα	CR	AVE
				2.5%	97.5%			
Attitude						0.73	0.80	0.55
AT1	3.08	1.41	0.85	0.83	0.87			
AT2	3.09	1.30	0.67	0.60	0.74			
AT3	3.39	1.27	0.84	0.81	0.87			
AT4	2.19	0.94	0.57	0.48	0.65			
Subjective Norm						0.76	0.82	0.80
SN1	2.61	1.31	0.93	0.92	0.94			
SN2	2.69	1.37	0.86	0.82	0.89			
Perceived Behavioural Control						0.66	0.70	0.59
PBC1	3.42	1.36	0.85	0.83	0.87			
PBC2	2.48	1.51	0.77	0.72	0.81			
PBC3	2.78	1.50	0.69	0.61	0.75			
Intention to consume edible offal						0.83	0.84	0.85
IN1	3.03	1.43	0.93	0.92	0.94			
IN2	2.79	1.45	0.92	0.90	0.93			
Food Neophobia						0.86	0.87	0.51
FN1	1.48	0.95	0.73	0.68	0.77			
FN2	1.50	0.94	0.58	0.51	0.65			
FN3	2.29	1.03	0.70	0.64	0.75			
FN4	1.76	1.13	0.72	0.67	0.76			
FN5	1.52	0.93	0.77	0.73	0.81			
FN6	1.46	1.13	0.73	0.67	0.77			
FN7	1.60	1.03	0.79	0.75	0.83			
FN8	1.30	1.03	0.68	0.63	0.73			
Food Disgust						0.63	0.88	0.51
FD1	36.27	31.18	0.58	0.46	0.67			
FD2	55.33	37.66	0.86	0.82	0.92			
FD3	74.34	31.41	0.51	0.38	0.61			
FD4	54.15	35.35	0.53	0.41	0.63			

Note: AT = attitude; SN = subjective norms; PBC = perceived behavioural control; IN = intention; FN = food neophobia; FD = food disgust. In parentheses are presented the *p*-values.

**Table 3:** Discriminant validity: heterotrait-monotrait (HTMT) ratio correlations.

	AT	SN	PCB	IN	FN
AT					
SN	0.66				
PCB	0.77	0.79			
IN	0.84	0.79	0.83		
FN	0.33	0.32	0.34	0.38	
FD	0.47	0.52	0.41	0.46	0.47

Note: AT = attitude; SN = subjective norms; PBC = perceived behavioural control; IN = intention; FN = food neophobia; FD = food Disgust.

**Table 4:** Path coefficient assessment.

	SRMR = 0.04		
Hypothesis			Results
	Dependent variable: IN	R^2^ adj = 0.65	
		Standardized β	
H1	ATT	0.337 **	Supported
H2	PCB	0.337 **	Supported
H3	SN	0.242 **	Supported
H7	FN	−0.080 *	Supported
	Dependent variable: AT	R^2^ adj = 0.23	
		Standardized β	
H4	FD	−0.466 **	Supported
	Dependent variable: SN	R^2^ adj = 0.23	
		Standardized β	
H5	FD	−0.475 **	Supported
	Dependent variable: PCB	R^2^ adj = 0.15	
		Standardized β	
H6	FD	−0.387 **	Supported
	Dependent variable: FD	R^2^ adj = 0.19	
		Standardized β	
H8	FN	0.398 **	Supported
	Age	−0.108 **	
	Regional Area	0.087 *	
	Gender	−0.085 *	
	Dependent variable: FN	R^2^ adj = 0.04	
		Standardized β	
	Income	−0.173 **	

Note: AT = attitude; SN = subjective norms; PBC = perceived behavioural control; IN = intention; FN = food neophobia; FD = food disgust. ** *p* < 0.01; * *p* < 0.05.

## Data Availability

All related data and methods are presented in this paper. Additional inquiries should be addressed to the corresponding author.

## Appendix A

**Pictures**					
**Picture**	**Description**	**Source**	**Domain**	**Disgust Scores**	
				**M**	**SD**
	Wrinkled tomatoes	(a)	Rotting food	36.26	31.38
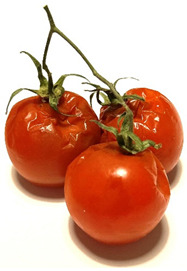
	Beef tongue	(a)	Animal flesh	55.33	37.66
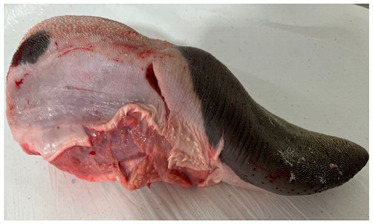
	Marmalade with mould	(a)	Mould	74.34	31.41
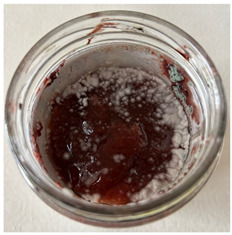
	Decaying banana	(a)	Decaying food	54.15	35.15
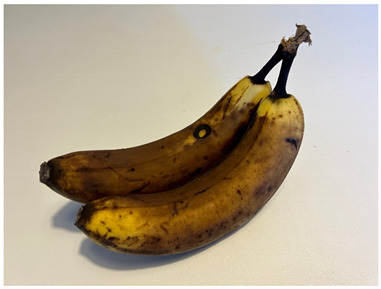
	Hands handling dough, rings, painted nails	(a)	Contamination	60.47	32.48
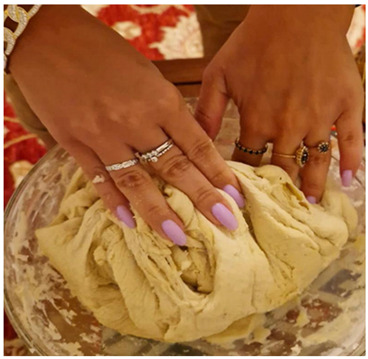
Note: (a) own pictures.					
